# Minimally invasive vs. open esophagectomy in patients with a BMI > 35: a NSQIP analysis

**DOI:** 10.1007/s00464-026-13074-z

**Published:** 2026-07-13

**Authors:** Sneha S. Alaparthi, Scott H. Koeneman, Olugbenga T. Okusanya, Tyler Grenda, Nathaniel R. Evans, Scott W. Cowan

**Affiliations:** 1 Division of Thoracic and Esophageal Surgery, Department of Surgery, Thomas Jefferson University Hospital, Philadelphia, PA 19107 USA; 2 Division of Biostatistics and Bioinformatics, Department of Pharmacology, Physiology and Cancer Biology, Sidney Kimmel Medical College, Philadelphia, PA 19107 USA; 3 Department of Surgery, Thomas Jefferson University Hospital, 211 South 9th Street Suite 300, Philadelphia, PA 19107 USA

**Keywords:** Esophagectomy, Minimally invasive, BMI

## Abstract

**Background:**

In this analysis, we aim to examine the outcomes of minimally invasive and open esophagectomy in patients with a BMI > 35.

**Study design:**

This is a retrospective cohort study utilizing the NSQIP Targeted Esophagectomy database from 2016 to 2023. We identified patients with a BMI > 35 who underwent minimally invasive esophagectomy (MIE) and open esophagectomy (OE) for malignancy. Primary outcome measures included morbidity, mortality, and incidence of anastomotic leak. Secondary measures included length of stay (LOS), unplanned return to OR, pneumonia, ventilator > 48 h, unplanned intubation, and SSI.

**Results:**

243 patients underwent MIE and 221 patients underwent OE. 30-day mortality was 2.87% in the MIE cohort and 4.32% in the open cohort [OR 1.53, 95% CI 0.45–5.45]. Overall morbidity was 41.1% in the MIE cohort vs 45.3% in OE [OR 1.18, 95% CI 0.80–1.74]. Anastomotic leak showed no significant difference between both cohorts [19% MIE vs 17% OE, OR 0.87, 95% CI 0.52–1.43]. LOS was significantly lower in the MIE cohort compared to OE (9.96 days vs. 11.31, 95% CI 2.69–0.02, *P* = 0.046). After logistic regression to adjust for confounding covariates, the estimated odds of experiencing each binary outcome, aside from pneumonia, were higher in the OE cohort. LOS increased by 1.41 days (95% CI 0.05–2.77) in the OE cohort after linear regression to adjust for confounding covariates.

**Conclusions:**

We demonstrate that MIE has comparable outcomes to OE in several measures in patients with a BMI > 35. We also found significantly improved LOS in the MIE cohort. Our data suggest that in this patient population, MIE may demonstrate a comparable safety profile to the traditional open approach.

**Supplementary Information:**

The online version contains supplementary material available at https://doi.org/10.1007/s00464-026-13074-z.

Esophageal cancer (EC) is the 11th most prevalent cancer and the 7th leading cause of cancer death worldwide [1–3]. Although worldwide squamous cell carcinoma (SCC) accounts for a majority of the cases, in the United States and western countries, adenocarcinoma has become the leading subtype [4]. Major risks for SCC include consumption of alcohol and smoking [5]. In adenocarcinoma, risks include smoking, obesity, gastroesophageal reflux disease (GERD), and Barrett’s esophagus [5]. The rising obesity rates in western populations may in turn contribute to gastroesophageal reflux disease (GERD) and Barrett’s esophagus [4]. Rates of obesity in addition have doubled since 1990, which has paralleled the rise in prevalence in AC [6]. Esophagectomy is a mainstay of treatment for early-stage cancer. While malignancy remains the most common indication for esophagectomy in the US, it is also performed for high-grade dysplasia and a small subset of benign conditions (i.e., end-stage achalasia, strictures) [2]. Noting this trend and the rise in obesity and paralleled EC, it is imperative to study the outcomes of esophagectomy in this patient population [7, 8]. Minimally invasive esophagectomy (MIE) has emerged as a safe and effective option over the course of the last decade [7]. There is a lack of data examining the safety profile of MIE in specific high risk. In this analysis, we aim to examine esophagectomy outcomes in patients with a BMI greater than 35, comparing open and minimally invasive approaches.

## Patients and methods

This is a retrospective cohort study utilizing the National Surgical Quality Improvement Program (NSQIP) Targeted Esophagectomy database from 2016 to 2023. We identified patients with a Body Mass Index (BMI) > 35 who underwent minimally invasive esophagectomy (MIE) and open esophagectomy (OE). Patients with a hybrid approach, conversion to open or unknown approach were excluded from the final analysis. Patients with unknown or nonmalignant pathologies were excluded from the analysis. Primary outcome measures included morbidity, mortality, and leak rate. Secondary measures included length of stay (LOS), unplanned return to operating room (OR), pneumonia, ventilator > 48 h, unplanned intubation, and surgical site infection (SSI). Outcomes were first compared between the MIE and OE cohorts in unadjusted analysis. Binary outcomes were compared using odds ratios with Wald-type confidence intervals. Length of stay, the lone numeric outcome, was compared using a mean difference by way of a *t*-test and *t*-intervals. To further analyze the outcomes while adjusting for potential confounding covariates, statistical modeling was employed. Logistic regression modeling was used to estimate odds ratios of experiencing binary outcomes between the MIE and OE groups while adjusting for American Society of Anesthesiologists (ASA) class, sex, diabetes, smoking status, history of severe COPD, history of heart failure within 30 days prior to operation, and functional health status. A Gaussian linear regression model was used to model the effect of open surgery on LOS as compared to MIE, while adjusting for the same covariates used in the logistic regression model. Data were analyzed using R version 4.3.1.

This study was reviewed by the Office of Human Research at Thomas Jefferson University and determined to be exempt from Institutional Review Board (IRB) review.

This study was conducted and reported in accordance with the STROBE checklist.

## Results

Out of total 8936 cases, 887 patients had a BMI > 35 (9.9%). After removing patients with nonmalignant or unknown pathologies, 724 patients remained. Those with a hybrid approach, conversion to open, or unknown approach were excluded, producing a final total of 464 patients eligible for inclusion in the study. 243 patients underwent MIE, and 221 patients underwent OE (Table 1). 354 patients were male (76.29%) and 110 female (23.71%). The average age of the full cohort was 61.2 years old, with a median of 62. The average BMI was found to be 39.69, with a median of 38.13. A majority of patients were White (358, 77.16%). Diabetes was present in 147 patients (31.68%). 75 patients were smokers (16.16%). In the functional health status measure, 460 patients were noted to be independent (99.14%), followed by 4 partially dependent (0.86%). 35 patients had ASA class 1–2 (7.56%). 428 patients had an ASA class of 3 + (92.44%). 423 patients had adenocarcinoma (91.16%). 13 patients had squamous cell carcinoma (2.80%), in addition to 12 patients with dysplasia (2.59%), and 16 designated as other malignant (3.45%).

**Table 1:** Demographics

Category	Variable	Full cohort (*N* = 464)	MIE (*N* = 243)	OE (*N* = 221)
Demographics	Male	354 (76.3%)	185 (76.1%)	169 (76.5%)
	Female	110 (23.7%)	58 (23.9%)	52 (23.5%)
	Age, years–mean (median)	61.2 (62.0)	61.8 (63.0)	60.5 (61.0)
	Height, inches–mean (median)	68.0 (69.0)	68.1 (69.0)	67.8 (69.0)
	Weight, lbs–mean (median)	261.5 (254.5)	261.6 (259.0)	261.3 (251.0)
	BMI, kg/m^2^–mean (median)	39.7 (38.1)	39.6 (38.0)	39.8 (38.2)
	White	358 (77.2%)	191 (78.6%)	167 (75.6%)
	Unknown/not reported	87 (18.8%)	43 (17.7%)	44 (19.9%)
	Black or African American	11 (2.4%)	5 (2.1%)	6 (2.7%)
	Asian	5 (1.1%)	3 (1.2%)	2 (0.9%)
	American Indian/Alaska native	2 (0.4%)	0 (0.0%)	2 (0.9%)
	Some other race	1 (0.2%)	1 (0.4%)	0 (0.0%)
	Hispanic ethnicity–yes	18 (3.9%)	11 (4.5%)	7 (3.2%)
	Hispanic ethnicity–—no	369 (79.5%)	192 (79.0%)	177 (80.1%)
Health history	Diabetes mellitus	147 (31.7%)	74 (30.5%)	73 (33.0%)
	Smoker	75 (16.2%)	31 (12.8%)	44 (19.9%)
	Functional status–independent	460 (99.1%)	240 (98.8%)	220 (99.5%)
	Functional status–partially dependent	4 (0.9%)	3 (1.2%)	1 (0.5%)
	Functional status–totally dependent	0 (0.0%)	0 (0.0%)	0 (0.0%)
	Ventilator dependent	0 (0.0%)	0 (0.0%)	0 (0.0%)
	Severe COPD	32 (6.9%)	20 (8.2%)	12 (5.4%)
	Heart failure	10 (2.2%)	5 (2.1%)	5 (2.3%)
	Hypertension w/ meds	294 (63.4%)	151 (62.1%)	143 (64.7%)
	Dialysis	0 (0.0%)	0 (0.0%)	0 (0.0%)
	Ascites	0 (0.0%)	0 (0.0%)	0 (0.0%)
	CHF	10 (2.2%)	5 (2.1%)	5 (2.3%)
	Renal failure	0 (0.0%)	0 (0.0%)	0 (0.0%)
	Immunosuppressive therapy	20 (4.3%)	11 (4.5%)	9 (4.1%)
	Preop transfusion	0 (0.0%)	0 (0.0%)	0 (0.0%)
	Preop sepsis	4 (0.9%)	2 (0.8%)	2 (0.9%)
	ASA Class 1–2	35 (7.6%)	18 (7.4%)	17 (7.7%)
	ASA Class 3 +	428 (92.4%)	225 (92.6%)	203 (92.3%)
Surgery priority	Emergent/urgent	3 (0.6%)	1 (0.4%)	2 (0.9%)
	Elective	456 (98.3%)	240 (98.8%)	216 (97.7%)
	Unknown	5 (1.1%)	2 (0.8%)	3 (1.4%)
Operative time	Minutes–mean (median)	395.4 (379.0)	444.5 (455.0)	341.5 (315.0)
Pathology	Adenocarcinoma	423 (91.2%)	226 (93.0%)	197 (89.1%)
	Dysplasia	12 (2.6%)	3 (1.2%)	9 (4.1%)
	Squamous cell carcinoma	13 (2.8%)	4 (1.6%)	9 (4.1%)
	Other-malignant	16 (3.4%)	10 (4.1%)	6 (2.7%)
Clinical stage	Stage 0	3 (0.6%)	0 (0%)	3 (1.4%)
	Stage 1	74 (15.9%)	31 (12.8%)	43 (19.5%)
	Stage 2	68 (14.7%)	35 (14.4%)	33 (14.9%)
	Stage 3	176 (37.9%)	96 (39.5%)	80 (36.2%)
	Stage 4	41 (8.8%)	25 (10.3%)	16 (7.2%)
	Stage unknown	102 (22.0%)	56 (23.0%)	46 (20.8%)

Data presented as *N* (%) for categorical variables and mean (median) for continuous variables

*COPD* Chronic obstructive pulmonary disease, *CHF* chronic heart failure

Among the MIE cohort, 185 patients were male (76.13%), and 58 were female (23.87%). The average age in this cohort was 61.8 and the average BMI was 39.58. 226 patients had a diagnosis of adenocarcinoma (93.00%), 3 had dysplasia (1.24%), 4 had squamous cell carcinoma (1.65%), and 10 had another malignancy (4.12%) (Table 1).

In the open cohort, 169 patients were male (76.47%), and 52 were female (23.53%). The average age in this cohort was 60.52 and the average BMI was 39.80. In this cohort, 197 patients had a diagnosis of adenocarcinoma (89.14%), 9 had squamous cell carcinoma (4.07%), 9 had dysplasia (4.07%), and 6 were designated as other malignant (2.72%) (Table 1).

**Fig. 1:**
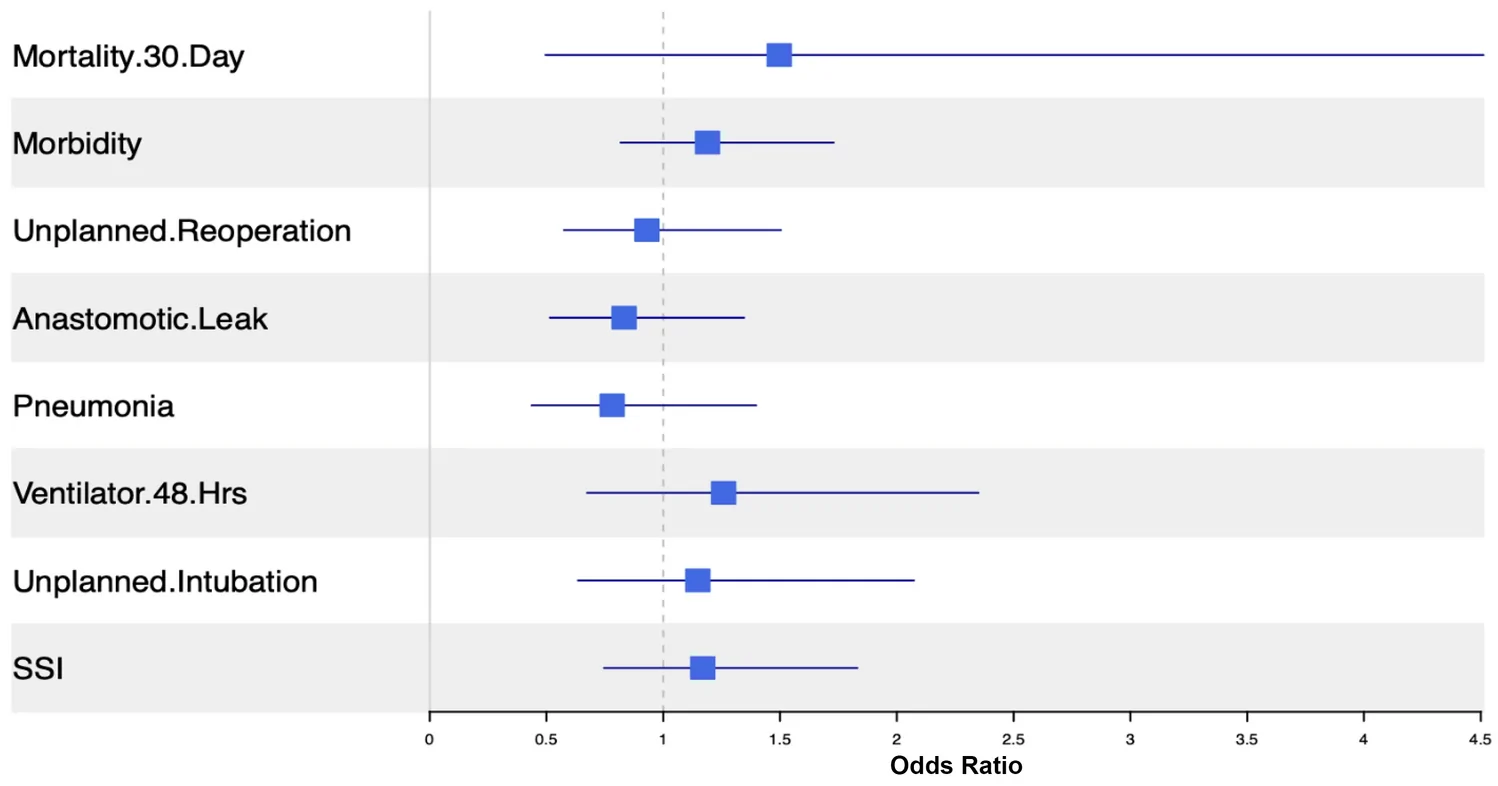
Model-adjusted ratios of outcomes

Overall, 30-day mortality was 2.87% in the MIE cohort and 4.32% in the open cohort [OR 1.53, 95% CI 0.66–6.64] (Table 2). Overall morbidity was 41.15% in the MIE cohort vs 45.25% in OE [OR 1.18, 95% CI 0.804–1.737]. There was no significant difference in anastomotic leak between both cohorts, 19.34% in MIE and 17.19% in OE [OR 0.866, 95% CI 0.52–1.43]. LOS was significantly lower in the MIE cohort compared to OE (9.96 days vs. 11.31, 95% CI −2.69 to 0.02, *P* = 0.046) (Table 2). Rates of unplanned return to the OR and pneumonia demonstrated no significant difference between both cohorts. No significant differences were observed for SSI, ventilator use or unplanned intubation. Operative time was an average of 444.5 min in the MIE cohort and 341.5 in the open cohort. The observed difference in operative times was 102/95 min, with a 95% CI of 78.4–127.5. After model-adjusted logistic regression, the odds of experiencing each outcome (aside from pneumonia) were increased in the OE cohort (Table 3, Fig. 1). Linear regression was used to adjust for covariates in measuring the effect between procedure type and LOS. This model estimated that LOS demonstrated a mean difference of 1.41 days between cohorts (95% CI 0.05–2.77).

**Table 2:** Unadjusted outcomes

Outcome	OE (*N* = 221)	MIE (*N* = 243)	Unadjusted OR (95% CI)
30-Day mortality	8/185 (4.3%)	6/209 (2.9%)	1.528 (0.455–5.450)
Overall morbidity	100/221 (45.3%)	100/243 (41.2%)	1.181 (0.804–1.737)
Unplanned reoperation	41/221 (18.6%)	46/243 (18.9%)	0.976 (0.594–1.598)
Anastomotic leak	38/221 (17.2%)	47/243 (19.3%)	0.866 (0.523–1.426)
Pneumonia	23/221 (10.4%)	32/243 (13.2%)	0.766 (0.413–1.404)
Ventilator > 48 h	26/221 (11.8%)	22/243 (9.1%)	1.339 (0.704–2.565)
Unplanned intubation	27/221 (12.2%)	25/243 (10.3%)	1.213 (0.653–2.260)
Surgical site infection	51/221 (23.1%)	50/243 (20.6%)	1.158 (0.727–1.845)
Length of stay, days (*P* = 0.046)	11.31	9.96	−1.354 (−2.686 to−0.022)

**Table 3:** Adjusted outcomes

Outcome	OE (*N* = 221)	MIE (*N* = 243)	Adjusted OR (95% CI)
30-Day mortality	8/185* (4.32%)	6/209 (2.87%)	1.497 (0.496–4.511)
Overall morbidity	100/221 (45.25%)	100/243 (41.15%)	1.19 (0.818–1.72998)
Unplanned reoperation	41/221 (18.55%)	46/243 (18.93%)	0.930 (0.576–1.503)
Anastomotic leak	38/221 (17.19%)	47/243 (19.34%)	0.832 (0.515–1.346)
Pneumonia	23/221 (10.41%)	32/243 (13.17%)	0.781 (0.437–1.398)
Ventilator > 48 h	26/221 (11.76%)	22/243 (9.05%)	1.258 (0.674–2.349)
Unplanned intubation	27/221 (12.22%)	25/243 (10.29%)	1.148 (0.635–2.074)
Surgical site infection	51/221 (23.08%)	50/243 (20.58%)	1.169 (0.747–1.831)

Patients with missing variables excluded

## Discussions

The impact of obesity on surgical approach and outcomes after esophagectomy remains incompletely studied. In this analysis of a national cohort, we observed that MIE has comparable outcomes to OE in several measures among patients with a BMI > 35. We additionally found significantly improved LOS among patients who underwent MIE. These results remained consistent when adjusting for several covariates. Although multiple outcome measures were not statistically significant, our data suggest that in patients with a BMI > 35, MIE may demonstrate a comparable safety profile to the traditional open approach.

A BMI > 35 was used as a cutoff as prior research has demonstrated an inflection point in outcomes at this level. Conroy et al. in 2023 performed a study of 4165 patients in the NSQIP targeted esophagectomy database. These patients were stratified by a BMI < or > 35 and the latter cohort was found to have significant higher anastomotic leak rates, longer operative times, and increased rate of SSI [8], This study additionally demonstrated that a BMI > 35 was an independent predictor of leak when esophagectomy was performed for malignancy or dysplasia [8]. A Society of Thoracic Surgeons (STS) database study in 2018 found a similar correlation when stratifying esophagectomy complications by BMI [9]. In this cohort, overweight (BMI 25–29.9) and obesity class I patients (BMI 30–34.9) had decreased risk for most complications when compared to normal, while patients with obese class II (BMI 35–39.9) and class III (BMI > 40) had increased risk [9]. These including any major complication, cardiovascular complication, and thromboembolic complication for a BMI > 35. For those with a BMI > 40, this included all of the previously listed complications in addition to pulmonary and infectious complications [9]. While there is no specific epidemiological data examining this subset of obesity in the US population, the current prevalence of class III obesity (BMI > 40) is approximately 9.4% [10]. Additionally, Ward et al. have predicted that approximately 1 in 4 adults will have severe obesity (BMI > 35) by 2030 (24.2%) [11]. As this population continues to grow, it is imperative to study outcomes among the highest risk patients with respect to esophagectomy.

A small but statistically significant decrease in LOS was observed in patients undergoing MIE (9.96 days vs 11.31). This is consistent with prior research. Specifically, Tohmasi et al. found that MIE patients had significantly shorter hospital stays compared to open (8 vs 11 days, *P* < 0.001). MIE in this study was associated with a 56% reduction in odds of prolonged hospitalization (OR 0.44). They additionally noted a significantly lower in-hospital mortality rate among MIE patients (2.7%) compared to OE (5.3%, *P* = 0.001) [12]. A meta-analysis performed in 2022 by Harriott et al. found an improving length of stay when comparing open, hybrid, and totally minimally invasive approaches. Median LOS in this population was found to be 14.1 days in the OE cohort, 12.5 days in the hybrid cohort, and 11.9 days in MIE (*P* = 0.003) [13]. While this study examined all patients, our data underscore this finding among patients with a BMI > 35. This suggests that patients in this specific population may also benefit from improved LOS with a minimally invasive approach.

Odds of anastomotic leak were not significantly different between MIE and OE cohorts (19.34% vs 17.19%), and this remained true after model-adjusted analysis. Multiple prior studies have examined the risks associated with leak in esophagectomy. Commonly cited variables include diabetes, old age, smoking status, frailty index, and type of esophagectomy [14, 15]. We adjusted for several similar metrics (DM, smoking status, functional status) in our model and found odds of leak still comparable. This metric is significant to patient outcomes, contributing significantly to morbidity and mortality. Schuchert et al. noted a morbidity of 70% vs 47% in patients who developed a leak, in addition to higher rates of stricture and need for postoperative dilation [16]. Anastomotic leak is also associated with a reduction in median overall survival, and disease-free survival [17]. Patients with a BMI > 35 have also demonstrated higher leak rates compared to those < 35 [8]. The prior study also noted that BMI > 35 was predictive of leak in a subgroup analysis of patients with malignancy/dysplasia [8]. These data highlight the importance of highlighting this specific metric among obese patients undergoing MIE compared to OE. Our study shows that in patients with a BMI > 35, MIE vs OE has comparable leak rates in adjusted and unadjusted analyses.

Minimally invasive esophagectomy in the obese patient has been studied in a limited capacity thus far. Prior research has shown that obesity has an impact on treatment approach in esophageal cancer. Sachdeva et al. found that in a review of the Society of Thoracic Surgeons database, obese and morbidly obese patients were more likely to undergo open procedures compared to patients with a normal BMI [18]. They additionally found higher rates of intraoperative conversion to open and longer operative times in this population [18]. A 2018 meta-analysis found that high BMI was associated with higher rates of wound infection, cardiovascular complications, and anastomotic leak [19]. A similar study by Wang et al. in 2019 found similar results, documenting higher risks of overall complications, leak, and thromboembolic complications in obese patients undergoing esophagectomy for cancer [20]. Among obese patients, some data have shown improved outcomes in MIE patients. A 2009 single institution study found that major complication rates were similar between obese and nonobese patients undergoing MIE; however, we found no difference in median ICU stay or LOS [21]. Our study examines these same metrics using a national database with a focus on comparison of MIE vs OE. We found no significant difference in 30-day mortality, overall morbidity, and unplanned return to operating room in patients with a BMI > 35 undergoing MIE or OE. Prior studies have also found higher rates of wound infection in this patient population [20]. We found SSI rates to be higher in the OE cohort; however, this was not statistically significant. Our data provide a promising window into the safety profile of MIE in obese patients. Further research is needed to understand how this information may affect clinical decision making and patient selection.

Our study has several limitations. Firstly, our data are limited to patients and centers participating in the NSQIP. While the volume of patients is large, the data may not be generalizable to the current population as this is a retrospective study which introduces inherent bias. We did not stratify by type of conduit for the purposes of this analysis, but this may have an independent effect on morbidity, leak rates, and other observed metrics. We had limited ethnic diversity in our cohort. The database is limited to patients from 2016 to 2023 and may not capture more historic or present-day changes in practice. The NSQIP database, while valuable for perioperative outcome analysis, does not provide oncologic variables (i.e., tumor stage, receipt of neoadjuvant therapy, pathologic findings, long-term or disease-free survival). This prevents the results from being interpreted within the context of oncologic safety. Additionally, approximately 20% of the study cohort had unknown clinical staging. While this may be an inherent limitation of this large-scale database, this affects the interpretation of our results within the context of this population. Our study was likely underpowered with a limited sample size given several metrics without statistical significance.

## Conclusions

In this retrospective cohort study, we found that patients with a BMI > 35 undergoing MIE or OE for esophageal malignancy had comparable outcomes in multiple measures. We additionally observed significantly improved LOS in patients who had MIE. No significant differences were found between cohorts when examining 30-day mortality, morbidity, and anastomotic leak. Given the rising rates of obesity in the US in addition to its strong correlation with esophageal cancer, this population warrants further research into safe surgical options. While prior research has focused on similar metrics, our data specifically examine MIE and OE in a subset of patients with a different risk profile for EC. Based on our study, MIE demonstrates no significant differences in safety profile to OE in obese patients when examining a national cohort. These data may be used to guide future research into this population and clinical decision making on an individualized basis.

## Supplementary Information

Below is the link to the electronic supplementary material.

Supplementary file1 (DOC 90 KB)
